# Carbon nanotubes-based PdM bimetallic catalysts through N_4_-system for efficient ethanol oxidation and hydrogen evolution reaction

**DOI:** 10.1038/s41598-019-47575-w

**Published:** 2019-07-30

**Authors:** Halima Begum, Mohammad Shamsuddin Ahmed, Dong-Weon Lee, Young-Bae Kim

**Affiliations:** Department of Mechanical Engineering, Chonnam National University, Gwangju, Republic of Korea

**Keywords:** Fuel cells, Electrocatalysis

## Abstract

Transitional metal-nitrogen-carbon system is a promising candidate to replace the Pt-based electrocatalyst due to its superior activity, durability and cost effectiveness. In this study, we have designed a simple strategy to fabricate carbon nanotubes-supported binary-nitrogen-carbon catalyst via wet-chemical method. Palladium and transitional metals (M, i.e. manganese cobalt and copper) nanoparticles are anchored through four-nitrogen system onto carbon nanotubes (denoted as PdM-N_4_/CNTs). This material has been used as bifunctional electrocatalyst for electrochemical ethanol oxidation reaction and hydrogen evolution reaction for the first time. The N_4_-linked nanoparticles onto carbon nanotubes plays a crucial role in intrinsic catalytic activity for both reactions in 1 M KOH electrolyte. Among three PdM-N_4_/CNTs catalysts, the PdMn-N_4_/CNTs catalyst exhibits higher catalytic activity in terms of current density, mass activity and stability compared to the benchmark Pt/C. The robust electrocatalysis are inherited from the better attachment of PdMn through N_4_-system onto carbon nanotubes, comparatively smaller particles formation with better dispersion and higher electrical conductivity.

## Introduction

Fast increasing energy demand and the deterioration of the environment from fossil fuels have triggered the research on renewable and sustainable clean energy sources, such as fuel cells (FCs) and water splitting cells^1–3^. In the meantime, FCs and water splitting cells have received intensive attention as the next-generation and clean energy sources due to their superior energy conversion efficiency, easy operation system, low reaction temperature, with negligible greenhouse gas production^4–7^. Ethanol oxidation reaction (EOR) and hydrogen evolution reaction (HER) are two important electrochemical reactions that take place in direct ethanol fuel cells (DEFCs) and water splitting cells, respectively^6,7^. Particularly, DEFCs, which use ethanol as fuel, are considered as forthcoming energy conversion devices because of nontoxic nature, abundance, easy to store, and cost effectiveness of ethanol^8,9^. However, the practical adoption of DEFCs technology is greatly hampered by the high cost, scarcity, low durability and higher CO-poisoning effect of the platinum (Pt)-based electrocatalysts^10–12^.

On the other hand, electrocatalytic water splitting is also an interesting research area because it’s a simple method to produce hydrogen gas with high purity which is using as a clean fuel nowadays^13–17^. Although, Pt-based materials are the most effective catalysts for HER but prohibitive cost and less durable catalysis make it nearly impossible to be used for large-amount of hydrogen generation^18–20^. Therefore, finding some alternative earth-abundant materials with high catalytic activity, low cost and long-term stable bifunctional electrocatalysts as the replacement of Pt-based catalysts that reduce the overpotential and increase the reaction rate are still anticipated for commercialization of FCs and large-amount of hydrogen production.

To design superior active catalysts, two important factors should be considered. First, increasing the number of active sites to enhance the mass transfer during the reaction process which are associated with the specific surface area and the homogeneous distribution of the dopant species. Second, the intrinsic nature of active sites to enhance the electrocatalytic activity which are determined by the catalyst composition and hence the accessible part of the active sites^21–23^. It has already proven that both two crucial factors are maintained in the transitional metal-nitrogen-carbon (M-N-C) catalyst system. These catalysts are involved with coordination between surface nitrogen and metal which enhance the mass transport properties for the electroactive molecules to active sites^24,25^. As a result, M-N-C catalyst system shows higher electrocatalysis for oxygen reduction reaction. Unfortunately, this system has not been intensively used in other electrochemical reactions, such as EOR and HER.

The palladium (Pd) is already been established as a promising metal source to replace Pt-based electrocatalysts because of its cheaper price, less poisoning effect from CO, greater practical stability and higher catalytic activity towards various electrochemical reactions including EOR and HER in alkaline media^12,25–28^. In addition, numerous transitional metals can be used as binary- and ternary-metallic catalysts with Pd for electrocatalytic reactions in alkaline media^29,30^.

Carbon nanotubes (CNTs) is an ideal supporting material for many electrocatalytic application due to its higher electrical conductivity, chemical stability and high specific surface area^11,31,32^. Density functional theory suggests that the Pd-fabricated CNTs could accelerate the catalytic activity towards various electrochemical reactions^33^. Recently, transitional metal (i.e. Mn, Co, Cu) phthalocyanines where a metal atom is coordinated by four nitrogen atoms (M-N_4_) are using for fabrication of carbon (i.e. CNTs, graphene)-based efficient electrochemical catalysts^34–37^. It has already proven that the M-N_4_-systems are regarded as superior electroactive catalysts for oxygen reduction^38,39^. Moreover, Petraki *et* *al*. are shown that the Mn-atom in manganese(II) phthalocyanine (MnPc) can make a deep electronic interaction with precious metals through 3d-electrons at the atomic level^40,41^.

Herein we have developed an electroactive and bifunctional material which derived from the Pd NPs grafted with the assistance of MnPc onto CNTs (denoted as PdMn-N_4_/CNTs) for EOR and HER electrocatalysis via simple wet-chemical method. The PdMn-N_4_/CNTs are rendering sufficient exposure of abundant active sites with the low cost and long-term operational as superior active catalyst. As a result, it shows better activity with more than two times higher electrochemical surface area (ECSA) and three times higher mass activity (MA) than the commercial Pt/C catalyst in EOR. On the other hand, PdMn-N_4_/CNTs also presents better catalytic behavior for HER by delivering a highest limited current density of 264 mA cm^−2^ with similar onset potential (*E*_onset_) and better stability than the Pt/C catalyst.

## Experimental

### Catalyst synthesis

In order to prepare Mn-N_4_/CNTs, a 30 mg of CNTs and 20 mg of MnPc was dispersed in 30 ml of water separately into two separate round-bottom flask. The MnPc solution was added into CNTs suspension slowly under stirring and kept for 6 h. Afterward, 15 mL of 20 mM K_2_PdCl_4_ solution (in water) was then added into the solution under gentle stirring. Subsequently, a cold 5 mL of 0.1 M NaBH_4_ solution was added slowly under gentle stirring for 1 h. Then the mixture was refluxed at 90 °C for one day under argon (Ar) atmosphere. With the elevated temperature, the metal precursors started to decompose, and the solution became dark. Finally, black PdMn-N_4_/CNTs was obtained after washing with DI-water and drying in a vacuum oven at 60 °C for 1 day. For comparison, PdMn/CNTs and Pd/CNTs were synthesized with the addition of MnCl_2_.(H_2_O)_4_ instead of MnPc and without MnPc, respectively. Also, PdCo-N_4_/CNTs and PdCu-N_4_/CNTs were synthesized with the same protocol by the addition of cobalt(ii) phthalocyanine (CoPc) and copper(ii) phthalocyanine (CuPc), respectively, instead of MnPc.

## Result and Discussions

### Surface morphology

**Figure 1:**
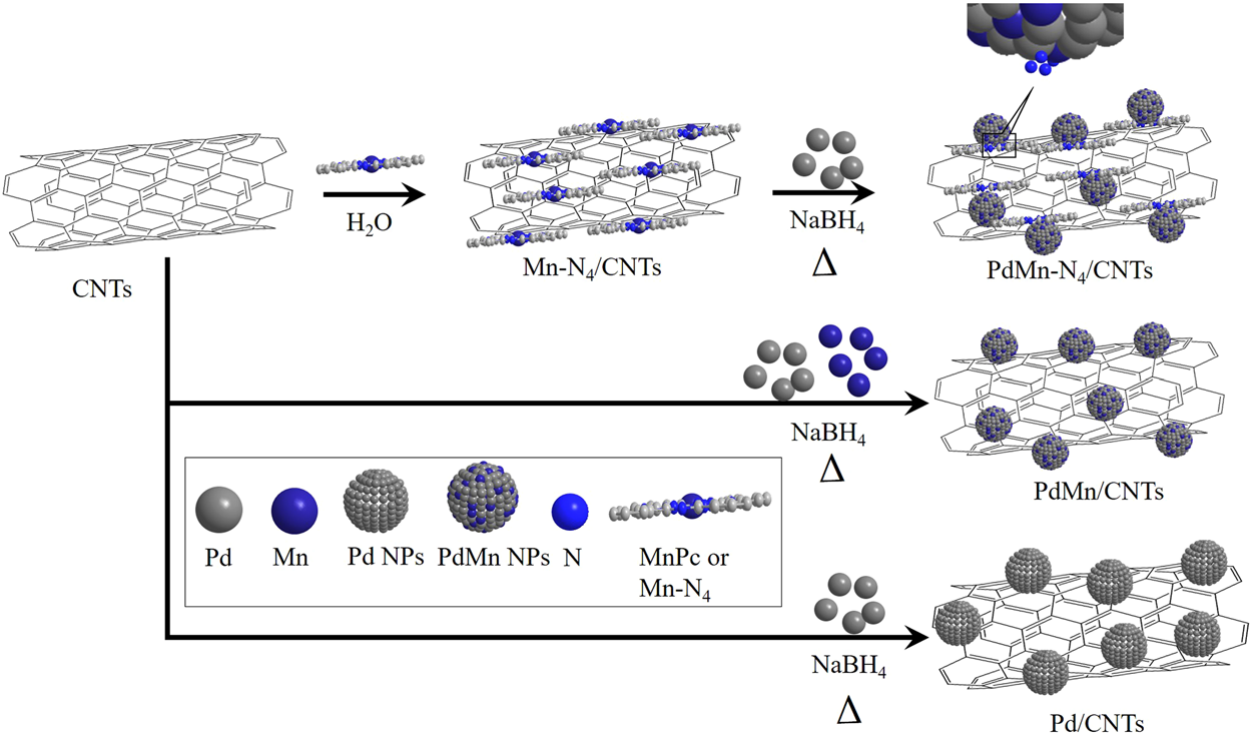
The schematic diagram of PdMn-N_4_/CNTs, PdMn/CNTs and Pd/CNTs synthesis.

**Figure 2:**
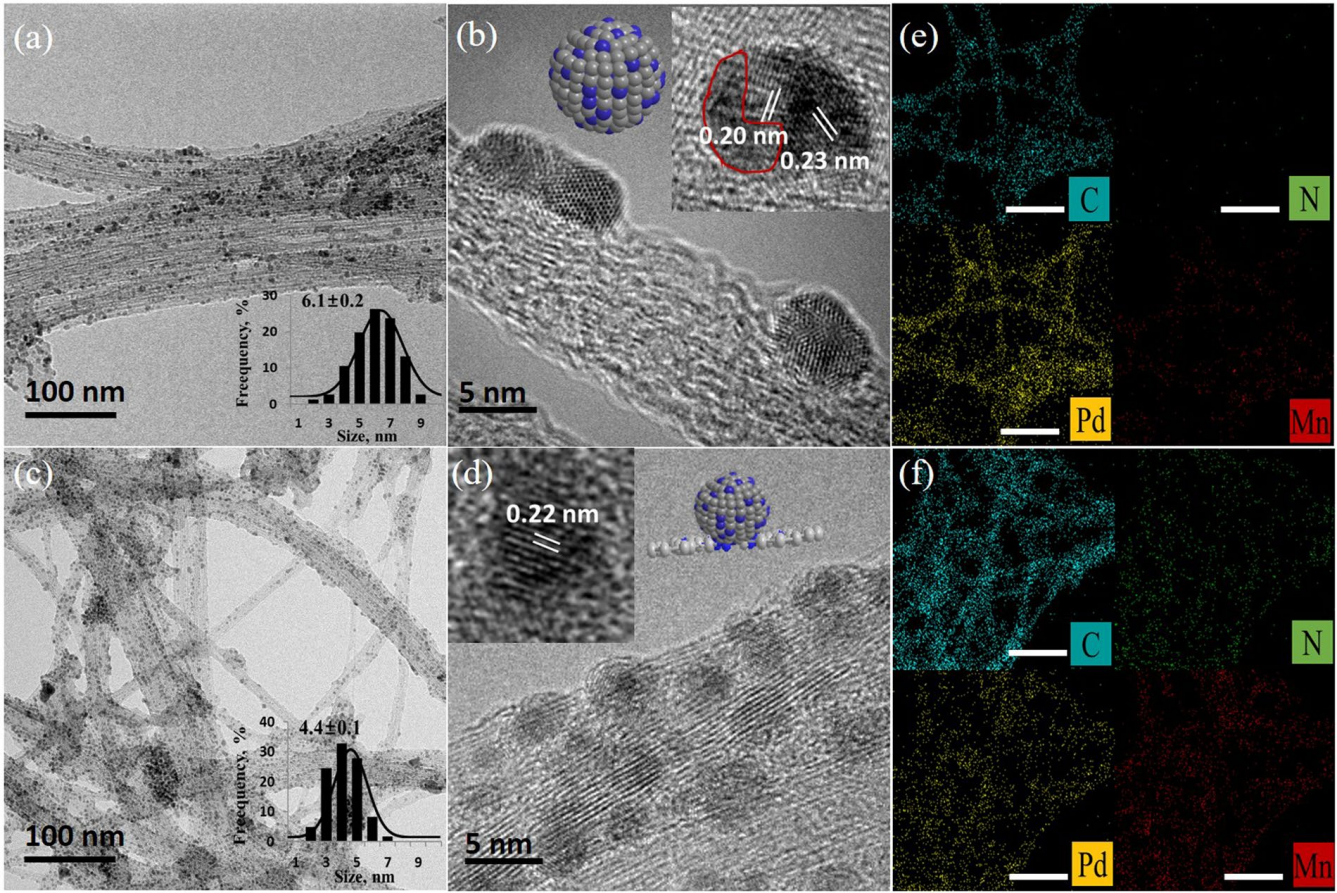
TEM images of PdMn/CNTs (**a**,**b**) PdMn-N_4_/CNTs (**c**,**d**) the mapping of PdMn/CNTs (**e**) and PdMn-N_4_/CNTs (**f**) samples in 100 nm scale, insets: a single PdMn NPs showing in corresponding HRTEM images with their simulated structures and red marking area is showing amorphous MnO_2_.

The MnPc was first anchored onto CNTs through π-π interaction and the K_2_PdCl_4_ solution was introduced with Mn-N_4_/CNTs in presence of NaBH_4_ aqueous solution (Fig. 1). The solution was then kept at 90 °C under gentle stirring for 24 h under Ar-atmosphere to form PdMn-N_4_/CNTs. The PdMn-N_4_/CNTs, PdMn/CNTs and Pd/CNTs were then characterized by transmission electron microscopy (TEM) analysis and the images of as-prepared composites are displaying in Fig. 2. The TEM image of PdMn/CNTs shows that the spherical-shaped PdMn NPs are grafted onto CNTs surface with fairly good dispersion (Fig. 2a) and the average size of the NPs is 6.1 nm (Fig. 2a inset). For better understanding, the high resolution TEM (HRTEM) image is also recorded which shows the PdMn NPs onto CNTs are anchored (Fig. 2b). The enlarged image shows the distinct lattice planes with *d*-spacing of 0.23 nm for Pd (111) plane and 0.20 for MnO_2_ (112) plane, in PdMn/CNTs sample. Also, the red line surrounded area indicated the amorphous manganese oxides^42^. For PdMn-N_4_/CNTs (Fig. 2c) consist of fine spherical PdMn NPs with homogeneous dispersion and the average size of the NPs is 4.4 nm (Fig. 2c inset). The HRTEM images of PdMn-N_4_/CNTs sample show PdMn NPs onto CNTs with excellent mono-dispersion (Figs 2d and S1a). The enlarged image shows the lattice plane with *d*-spacing of 0.22 nm which is the usual pure (111) plane of fcc Pd^15^. The *d*-spacing of PdMn NPs in PdMn/CNTs sample is little bigger due to the substitution of Pd with Mn atoms leads to the expansion of the Pd lattice^10^. The mapping analysis of PdMn/CNTs and PdMn-N_4_/CNTs samples are also shown in Fig. 2e,f, respectively. At a glance, it shows that the significant amount of nitrogen is present in PdMn-N_4_/CNTs, with homogeneous dispersion of Pd and Mn onto whole samples. Also, the energy dispersive spectroscopy (EDS) shows the amount of Mn is little higher in PdMn-N_4_/CNTs than the PdMn/CNTs (Fig. S1b). This is probably due to the free MnPc onto the CNTs surface. For comparison, the morphology of Pd/CNTs is also investigated by TEM and mapping (Fig. S2). which reveals that the Pd NPs is grafted with good dispersion onto CNTs. The TEM analysis is signifying the identical preparation method of all three catalysts.

### Characterization

**Figure 3:**
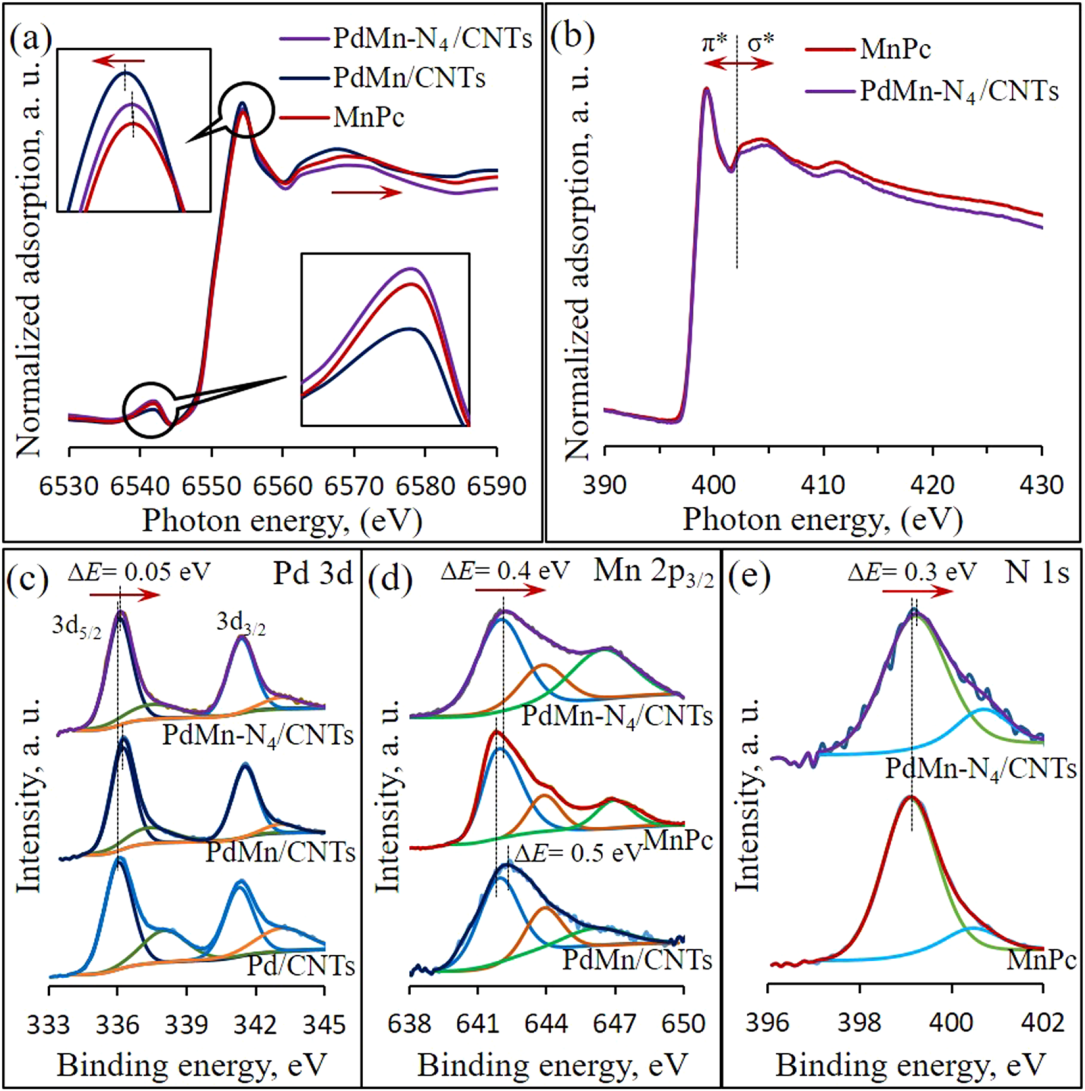
XAS spectra at Mn *K*-edge for PdMn/CNTs and PdMn-N_4_/CNTs (**a**) N *K*-edge for PdMn-N_4_/CNTs (**b**) core level of Pd 3d XPS spectra of Pd/CNTs, PdMn/CNTs and PdMn-N_4_/CNTs (**c**) Mn 2p XPS spectra of PdMn/CNTs and PdMn-N_4_/CNTs (**d**) and core level of N 1s XPS spectra of PdMn-N_4_/CNTs. (**e**) XAS and XPS data of pure (as purchased) MnPc is used for comparison.

Synchrotron X-ray absorption spectroscopy (XAS) was used to analyze the atomic structure of as prepared samples. The main absorption peaks in the Mn *K*-edge XAS spectra of PdMn-N_4_/CNTs was resembling feature to pure MnPc, in Fig. 3a. The lower inset shows the expanded pre-edge region which related to the local geometry of atomic sites. The slightly increased relative intensity of PdMn-N_4_/CNTs than the MnPc suggesting the similar coordination geometry of Mn sites in both samples^43^ and largely decreased relative intensity was probably due to Pd incorporation with Mn. Also, the intensity of oscillation hump in post-edge region elucidate the ordering extent of local atomic structure. Shown by similar inflection position, the metallic characteristics of Mn atoms in PdMn-N_4_/CNTs and MnPc samples are evident. The intensity of oscillation hump was higher at PdMn/CNTs than MnPc and PdMn-N_4_/CNTs (upper inset) which indicating the higher oxygen chemisorption by PdMn NPs in PdMn/CNTs^44^. Moreover, the higher peak shift in the negative direction compared to MnPc at PdMn/CNTs probably due to the higher degree of alloy formation in Pd and Mn, and very less in PdMn-N_4_/CNTs probably due to the coexistence. The N *K*-edge spectra of MnPc and PdMn-N_4_/CNTs are shown in Fig. 3b and there was no any significant change in the π* region, which indicated that no significant change in the coordination between Mn and N in both samples^45^.

The crystalline structure of all Pd-containing catalysts are investigated by X-ray powder diffraction (XRD) as shown in Fig. S4a. The XRD patterns of Pd/CNTs, PdMn/CNTs and PdMn-N_4_/CNTs reveal their fcc structures. Typically peaks at around 2*θ* = 40.1°, 46.7°, 68.3°, 81.9° and 86.1° are allocated to the crystalline Pd(111), Pd(200), Pd(220), Pd(311) and Pd(222) plans, respectively^46^, along with the peak at 2*θ* = 25.05° which is corresponding to the C(002) plan^47,48^. The PdMn/CNTs sample is displaying many other peaks which signifying the presence of amorphous MnO_2_ in the PdMn NPs^49^. However, the PdMn-N_4_/CNTs sample shows few peaks which representing MnO_2_. The significant blue-shift is observed from PdMn/CNTs sample (Δ2*θ* = 0.3). Whereas a little blue-shift is observed from PdMn-N_4_/CNTs sample (Δ2*θ* = 0.03) compared to the Pd(111) plan of Pd/CNTs (Fig. S3). Those findings indicate that Mn-atom has the highest entrance/interaction into the Pd lattice in PdMn/CNTs sample and less into the PdMn-N_4_/CNTs sample, probably due to free Mn addition (MnCl_2_) into PdMn/CNTs and coordinated Mn in MnPc (Mn-N_4_) into PdMn-N_4_/CNTs^50,51^. This result agrees well with the lattice *d*-spacing data using HRTEM.

X-ray photoelectron spectroscopy (XPS) technique is used to characterize the prepared samples to investigate insight into the composite formation as well as the elemental analysis. In Fig. S4b, two common peaks appeared in XPS spectra of all samples at ~284 eV and ~532 eV which signifying the presence of C and O elements, respectively^52,53^. The Mn (~640 eV) with N (~400 eV) and Pd (~335 eV) indicating peaks are appeared in the MnPc and Pd containing samples. The C and O ratio (C/O, at%) is then calculated in between 8.9 to 9.7. The amount of Pd is numerically determined as 10.3, 10.2 and 10.1 wt% in Pd/CNTs, PdMn/CNTs and PdMn-N_4_/CNTs, respectively. The amount of Pd is further confirmed by inductively coupled plasma with optical emission spectroscopy (ICP-OES) analysis and the result was slightly varied (10.31, 10.25 and 10.1 wt%, respectively) from XPS analysis.

The comparative Pd 3d XPS spectra of Pd/CNTs, PdMn/CNTs and PdMn-N_4_/CNTs are shown in Fig. 3c. All Pd 3d spectra present a doublet consisting of two bands at ~336 and ~340 eV which are assigned to the Pd 3d_5/2_ and Pd 3d_3/2_ spin-orbital doublets, respectively, and signifying that all Pd in those samples are mostly in Pd^0^ state. The Pd 3d_5/2_ peaks of PdMn/CNTs (336.22 eV) and PdMn-N_4_/CNTs (336.15 eV) samples are red-shifted than that of Pd/CNTs (336.1 eV) as can be seen in Fig. 3c. There is an evident positive shift (Δ*E* = 0.12 eV and 0.05 eV, respectively) in the binding energy for those samples, implying that higher charge interaction and synergistic effect between Pd and Mn^54^. In addition, the core level of Mn 2p XPS spectra of MnPc, PdMn/CNTs and PdMn-N_4_/CNTs exhibit two peaks at 642.01 eV and 653.6 eV, which can be ascribed to the Mn 2p_3/2_ and Mn 2p_1/2_ spin-orbital doublets, respectively (Fig. 3d). The Mn 2p_3/2_ shows a clear red-shift for PdMn/CNTs (0.2 eV) and PdMn-N_4_/CNTs (0.1 eV) compare to MnPc upon addition of Pd in presence of CNTs. Moreover, the core level of N 1s of MnPc and PdMn-N_4_/CNTs are presented in Fig. 3e. The N 1s spectrum of MnPc shows two peaks at 399.1 and 400.5 eV. By comparison, two peaks of PdMn-N_4_/CNTs are also shifted to 399.25 and 400.7 eV, respectively. Thus, the outcome of XPS analysis is that the mild red-shift in PdMn-N_4_/CNTs spectrum than Pd/CNT and MnPc in both Pd 3d and Mn 2p spectra, respectively, is probably due to the coordination between PdMn and N_4_-system which actually reduce the charge interaction between Pd and Mn. This result is well consistent with the XRD spectra shown in Fig. S4a and Raman spectra shown in Fig. S3. The red-shift in N 1s spectrum of PdMn-N_4_/CNTs than MnPc which is due to the chemical environment change arising from the electronic interaction between Pd and Mn in MnPc^55^ as demonstrated in Fig. 1.

### Electrochemical EOR

**Figure 4:**
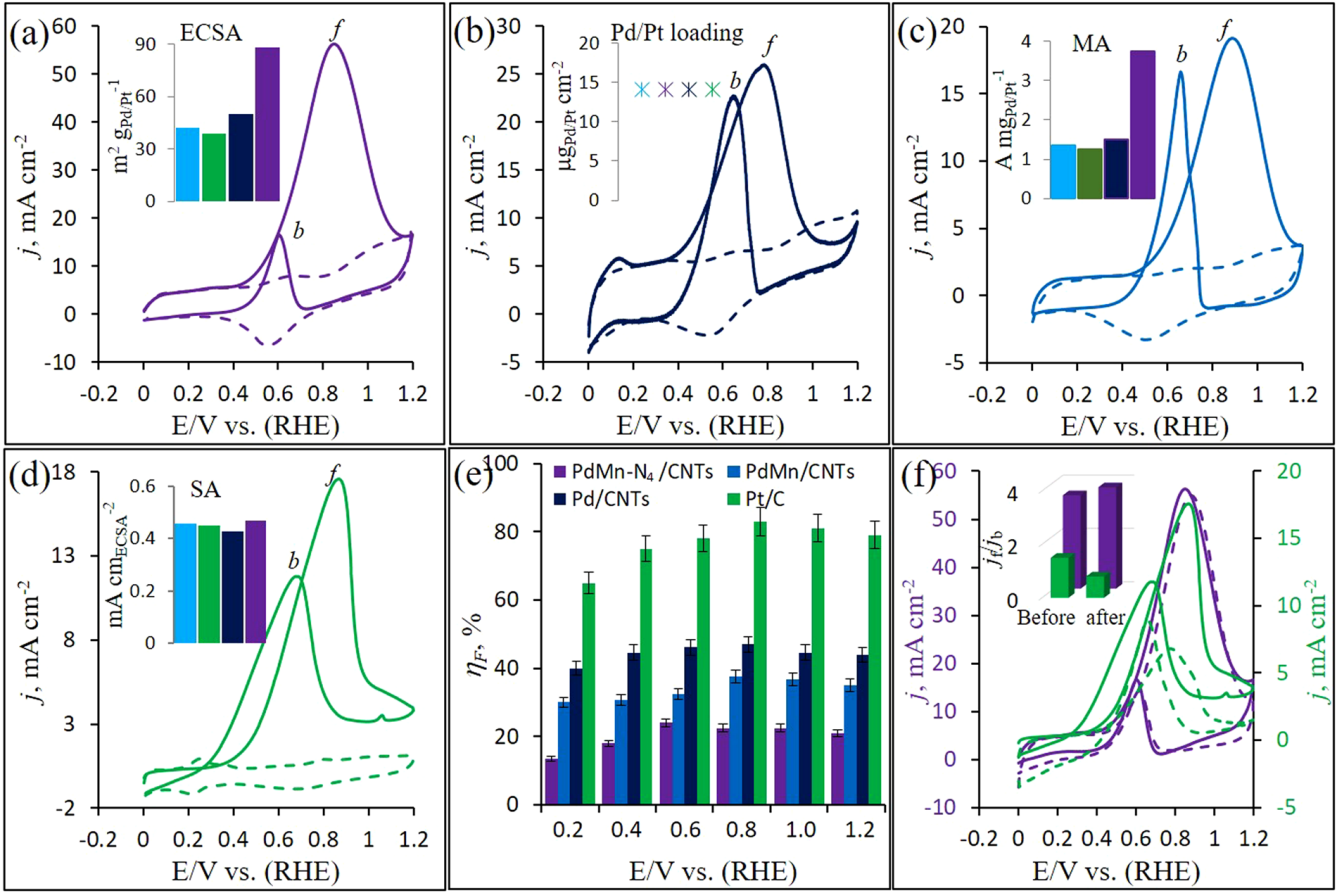
CVs recorded in Ar-saturated 1 M KOH electrolyte without (dotted lines) and with 2 M ethanol (solid likes) at a scan rate of 50 mV s^−1^ on PdMn-N_4_/CNTs (**a**) PdMn/CNTs (**b**) Pd/CNTs (**c**) and Pt/C (**d**) calculated faradaic efficiency for all electrodes (**e**) and CVs recorded before (solid lines) and after (dotted lines) 300 potential cycles at 50 mV s^−1^ scan rate on PdMn-N_4_/CNTs and Pt/C electrodes in the same solution containing 2 M ethanol (**f**) insets: the column chart of ECSA (**a**) metal-mass loading (**b**) MA (**c**) SA (**d**) for all tested electrodes and the *j*_*f*_/*j*_*b*_ of PdMn-N_4_/CNTs and Pt/C before and after 300 potential cycles (**f**).

At first the electrochemical behavior and EOR activity on PdM-N_4_/CNTs, PdMn/CNTs, Pd/CNTs and Pt/C have investigated using CVs in Ar-pursed 1 M KOH solution without (dotted lines) and with (solid lines) 2 M ethanol which are displayed in Fig. 4. At the reverse scan in blank CVs (dotted lines) from all Pd containing samples, a prominent PdO reduction peak is appeared in between −0.5 to −0.6 V. Also, the similar behavior (PtO reduction peak is appeared at −0.67 V) observed from Pt/C sample. The PdO reduction peak potential is shifted towards positive direction at PdMn-N_4_/CNTs and PdMn/CNTs catalysts compared to Pd/CNTs (Fig. S5). This distinct peak potential shifting behavior on PdMn-N_4_/CNTs and PdMn/CNTs catalysts indicate that the electronic structure has changed on the surface of these catalysts. The is due to the strong electronic interaction between Pd and Mn and good attachment with CNTs via N_4_-system in PdMn-N_4_/CNTs, and direct alloying with Mn in PdMn/CNTs catalyst^56^. The difference of double layer thickness is probably due to the variation of charging current. However, the ECSA for all catalysts has calculated using Coulombic charge (*Q*) which produce from the reduction of PdO or PtO^57,58^. The values of the ECSA are summarized in Fig. 4a inset and the highest value is obtained from PdMn-N_4_/CNTs among all catalysts which is 2.2 magnitudes higher than the Pt/C. Indicating higher degree of Pd-active site utilization on the PdMn-N_4_/CNTs electrode surface. This is probably due to the homogeneous dispersion and smaller size of NPs (while shape of NPs in all prepared samples are identically same) which strongly attached onto CNTs with the assistance of N_4_-system. Although, the preparation method was identically same with same amount of Pd-metal (Pt for Pt/C) loading (14.15 μg cm^−2^) on all electrodes (Fig. 4b inset).

The electrochemical ethanol oxidation on the above mentioned catalysts in alkaline medium is investigated using CVs technique in presence of 2 M ethanol (solid lines in Fig. 4a–d). All CV curves are shown two well-defined peaks which signifying EOR at all electrodes. Both two peaks are labeled as *f*-peak in the forward sweep and *b*-peak in the backward sweep, respectively. The intensity of *f*-peak is associated with the oxidation of freshly chemisorbed EtOH and the intensity of *b*-peak signifies the poisoning effect from non-oxidized carbonaceous species which produced during forward scan^59^. Among four EOR CV curves in Fig. 4, the highest current density is recorded at PdMn-N_4_/CNTs electrode (56.3 mA cm^−2^) which is about three times higher than that of Pt/C (17.5 mA cm^−2^). Also, PdMn/CNTs (25.6 mA cm^−2^) and Pd/CNTs (19.1 mA cm^−2^) produced much lower current density compare to PdMn-N_4_/CNTs. The ratio of *f*-peak intensity and *b*-peak intensity (*j*_*f*_/*j*_*b*_) is an indication of poisoning tolerance to the carbonaceous species at Pd-surface in a catalyst^58,60^. The higher *j*_*f*_/*j*_*b*_ is attributed to higher efficient electrooxidation of ethanol and less accumulation of carbonaceous species at the Pd-active site. The *j*_*f*_/*j*_*b*_ is determined by the corresponding CV curves and the value is calculated for PdMn-N_4_/CNTs electrode (3.3) which is notably higher than PdMn/CNTs (1.3) and Pd/CNTs (1.15) catalysts, signifying that the Pd-active site in PdMn-N_4_/CNTs catalyst is less affected by poisoning effect during EOR.

In addition, to compare the electrocatalytic activity towards EOR per loaded metal mass, the Pt- or Pd-mass (mass activity, MA) and ECSA (specific activity, SA) normalized current density are displayed in Fig. 4c,d insets, respectively. The PdMn-N_4_/CNTs catalyst provide the highest MA (3.74 A ${{\rm{mg}}}_{Pd}^{-1}$) at *j*_*f*_ which is 2.5, 2.8 and 3.0 times greater than those of PdMn/CNTs (1.5 A ${{\rm{mg}}}_{Pd}^{-1}$), Pd/CNTs (1.35 A ${{\rm{mg}}}_{Pd}^{-1}$) and Pt/C (1.24 A ${{\rm{mg}}}_{Pt}^{-1}$), respectively, as shown in Fig. 4c inset. This is consistent with the higher SA for EOR at PdMn-N_4_/CNTs catalyst (0.6 mA $c{m}_{ECSA}^{-2}$) than those of PdMn/CNTs, Pd/CNTs and Pt/C catalysts as shown in Fig. 4d inset, signifying much more favorable EOR on the PdMn-N_4_/CNTs electrode. Considering same metal-mass loading and identical preparation method with similar morphology, much higher electrocatalytic activity per metal-mass is observed at PdMn-N_4_/CNTs electrode which might be resultant from better ECSA, better attachment of PdMn NPs through N_4_-system and binary effect.

For comparison, the as-prepared PdCu-N_4_/CNTs and PdCo-N_4_/CNTs catalysts are employed for EOR in the same conditions and the EOR response from those catalysts is displayed in Fig. S6a. The EOR at those catalysts are lower than that of PdMn-N_4_/CNTs. This is probably due to the lower ECSA 65 and 53 m^2^ ${{\rm{g}}}_{Pd}^{-1}$, for PdCo-N_4_/CNTs and PdCu-N_4_/CNTs, respectively. Further CV curves are recorded in presence of 1 to 5 M ethanol in 1 M KOH for PdMn-N_4_/CNTs (Fig. S6b), PdCu-N_4_/CNTs (Fig. S6c) and PdCo-N_4_/CNTs catalysts (Fig. S6d). Here some important comparison is made. First, the *f*-peaks are proportionally increased with the increasing ethanol concentration (*C*_*EtOH*_) which indicating the ethanol dissociation and EOR catalysis are independent in any *C*_*EtOH*_ level at all three electrodes (corresponding Fig. S6 inset-i). Second, the *j*_*f*_/*j*_*b*_ is linear as the function of *C*_*EtOH*_ at PdMn-N_4_/CNTs electrode than those of PdCu-N_4_/CNTs and PdCo-N_4_/CNTs catalysts (corresponding Fig. S6 inset-ii) which indicates the ethanol oxidation and the oxidation of carbonaceous intermediates are formed in the same ratio at the PdMn-N_4_/CNTs catalyst^12^. Therefore, the better EOR catalysis is conducted at PdMn-N_4_/CNTs electrode than those of PdCu-N_4_/CNTs and PdCo-N_4_/CNTs catalysts.

### EOR kinetics

For understanding the EOR kinetics and major product at electrocatalyst surface, the faradaic efficiency (*η*_*F*_) can be calculated from the average number of electron transferred per ethanol molecule (*n*_*av*_). As it is known that the 12 electron (*e*^−^) involved EOR produces CO_2_ which shows higher *η*_*F*_ and 4*e*^−^ or 2*e*^−^ involved EOR produces acetic acid or acetaldehyde which shows lower *η*_*F*_^15,61^. The *η*_*F*_ is calculated from the Eq. (1) for all catalysts^62^ and is shown in Fig. 4e. The *η*_*F*_ is derived much lower at the PdMn-N_4_/CNTs catalyst than those on all other catalysts. This signifies the higher yield of acetaldehyde and/or acetate with less production of CO_2_ at the PdMn-N_4_/CNTs catalyst. However, the higher CO_2_ production is indicated at the Pt/C electrode during ethanol oxidation in our experiment which is similar to the other report^61^.

1 $${\eta }_{F}=\frac{{n}_{av}}{12}=\sum \frac{{n}_{i}{f}_{i}}{12}$$

where, *n*_*i*_ is the number of electrons transferred to product *i* and *f*_*i*_ is the fraction of ethanol converted to product *i*. All Tafel slope are nearly same 67.1, 67.7, 67.5 and 98.3 mV dec^−1^ for PdMn-N_4_/CNTs, Pd/CNTs, Pt/C and PdMn/CNTs catalysts, respectively (Fig. S7). The lower Tafel slope is attributed to the faster charge-transfer kinetics which facile to the electrochemical EOR process at catalyst surface^10^. Thus, faster charge-transfer during EOR is observed at PdMn-N_4_/CNTs catalyst surface.

The long-term stability of the catalyst is another one important factor that applies in practical application of DEFCs. The stability of the PdMn-N_4_/CNTs catalyst is compared with Pt/C catalyst using CV technique in 1 M KOH containing with 2 M ethanol as shown on Fig. 4f. The Fig. 4f demonstrates the current density is reduced slowly at PdMn-N_4_/CNTs electrode. After 300 CV cycles, PdMn-N_4_/CNTs electrode maintained 95% of the initial current density. After same CV cycles with same Pt-mass loading, however, Pt/C maintained 62% of the initial current density. Moreover, the *j*_*f*_/*j*_*b*_ is increased at 3.8 from 3.5 at PdMn-N_4_/CNTs electrode while decreased at 0.8 from 1.5 at Pt/C electrode (Fig. 4f inset). This is due to faster EOR kinetics and less active site blocking possess with a remarkable tolerance to poisoning by carbonaceous intermediates including CO^63^. These observations indicate the PdMn-N_4_/CNTs catalyst is much higher stabile for EOR in DEFCs.

### Electrochemical HER

**Figure 5:**
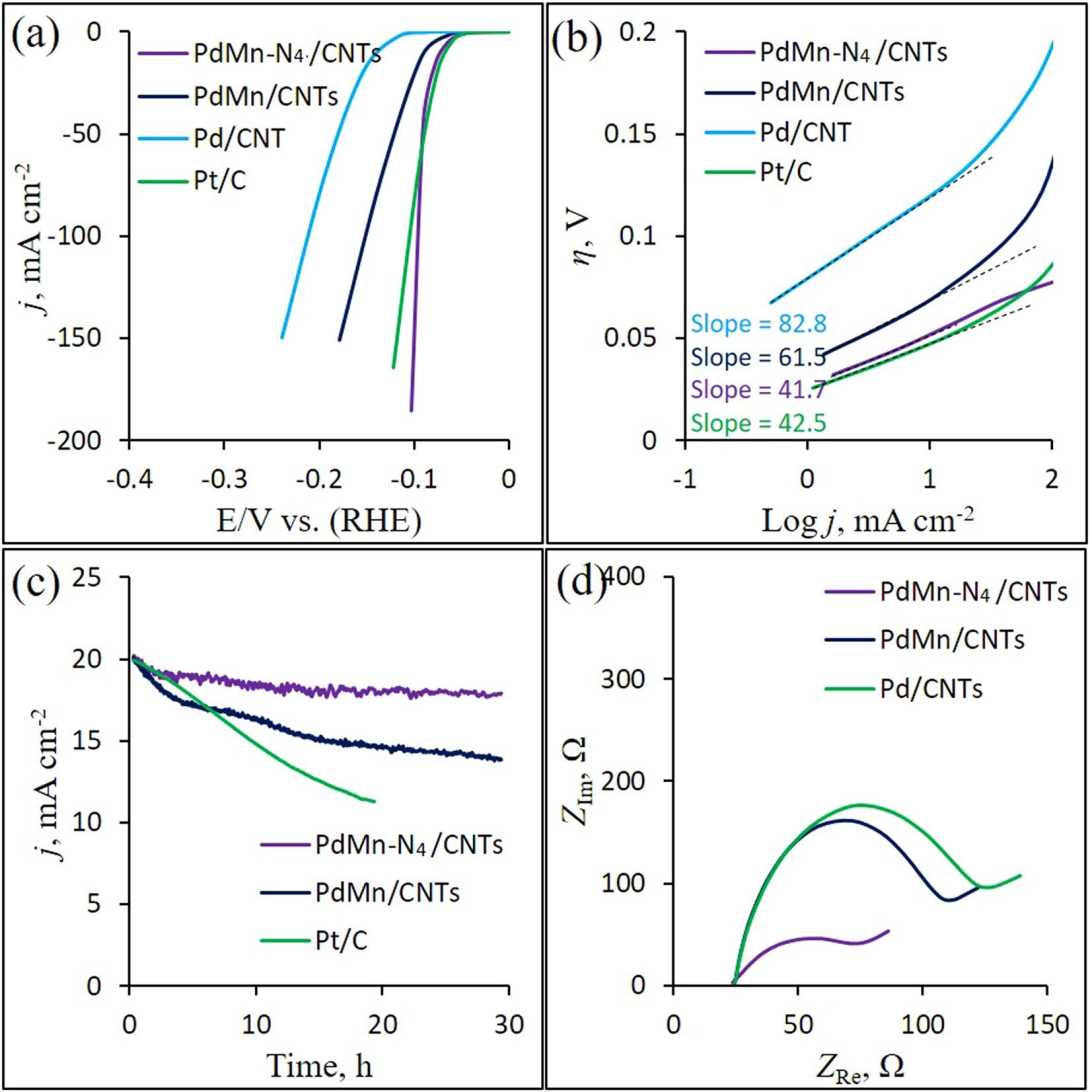
HER polarization curves at a scan rate of 5 mV s^−1^ (**a**) and the corresponding Tafel plots (**b**) of PdMn-N_4_/CNTs, PdMn/CNTs, Pd/CNTs and Pt/C electrodes in 1 M KOH solution, the *i* − *t* curves for stability test on PdMn-N_4_/CNTs, PdMn/CNTs and Pt/C electrodes at constant *η*_*j*20_ (80, 100 and 75 mV, respectively) for ~30 hours in the same solution (**c**) and EIS Nyquist plots of those electrodes (**d**).

To understand the bifunctional activity of prepared catalysts, the as prepared catalysts are employed for electrochemical HER. The electrochemical hydrogen evolution performance and stability of PdM-N_4_/CNTs, PdMn/CNTs, Pd/CNTs and Pt/C catalysts are evaluated under the same condition (1 M KOH) and the resulting curves are displayed in Fig. 5a. As illustrate in Fig. 5a, the HER curve from PdMn-N_4_/CNTs catalyst shows the highest HER performance with the *E*_onset_ of 35 mV and a overpotential at 10 mA cm^−2^ (*η*_*j*10_) of 71 mV which is comparable to the Pt/C (*E*_onset_ of 35 mV with *η*_*j*10_ of 65 mV), while the as-prepared PdMn/CNTs and Pd/CNTs perform comparatively poor HER activity (*E*_onset_ of 45 mV with *η*_*j*10_ of 90 mV and *E*_onset_ of 80 mV with *η*_*j*10_ of 137 mV, respectively). Important observation is that when the current density increases higher than 60 mA cm^−2^, the overpotential of PdMn-N_4_/CNTs catalyst is even lower than the Pt/C, which indicates that the PdMn-N_4_/CNTs catalyst is better active as well as stable for HER in the alkaline electrolyte^64^. Also, the HER activity of PdMn-N_4_/CNTs catalyst is comparable to many other electrocatalysts^15,17,54^. The higher HER performance from PdMn-N_4_/CNTs catalyst might be resultant from three unique factors. First, the PdMn NPs has large interlayer spacing at nanoscale that allows to rich density dispersing of HER active sites on it’s surface. Second, the PdMn NPs grafted via N_4_-system onto CNTs which enhances electrical conductivity. Third, higher ECSA that allows the Pd-active site utilization with higher degree. The turnover frequency (TOF) is calculated using Brunauer-Emmett-Teller (BET) surface area data in Fig. S8^65^. The HER at PdMn-N_4_/CNTs catalyst in the alkaline electrolyte was with TOF of 3.1 s^−1^ at 80 mV @ *η*_*j*20_. The calculated TOF of HER at PdMn/CNTs and Pd/CNTs was 2.6 and 1.05 s^−1^ at *η*_*j*20_ = 100 and 152 mV, respectively. Thus, these three distinct features guarantee the remarkable HER activity at PdMn-N_4_/CNTs catalyst.

For the HER catalysts, the Tafel slope is generally regarded as the indicator of rate determining step (RDS) of HER process. Three primitive steps in the HER process are expressed as below^15,66,67^:

1. ~120 mV dec^−1^ Tafel slope indicates the Volmer reaction: H_2_O + e^−^ → H_ads_ + HO^−^.
2. ~40 mV dec^−1^ Tafel slope indicates the Heyrovsky reaction: H_2_O + e^−^ + H_ads_ → H_2_ + HO^−^.
3. ~30 + mV dec^−1^ Tafel slope indicates the Tafel reaction: H_ads_ + H_ads_ → H_2_.

In Fig. 5b, the Pt/C displays a Tafel slope of 41.7 mV dec^−1^, which is close to the value of other reported Pt/C^68^ and indicates the RDS is hydrogen desorption according to the Volmer–Heyrovsky mechanism. Similarly, the PdMn-N_4_/CNTs exhibits a Tafel slope of 42.5 mV dec^−1^, suggesting that the HER catalytic process is dominated by the Volmer–Heyrovsky mechanism and the RDS is electrochemical hydrogen desorption. However, The PdMn/CNTs and Pd/CNTs possess Tafel slopes of 61.5 and 82.8 mV dec^−1^ which belong to the more complicated HER mechanism. The smaller Tafel slope of PdMn-N_4_/CNTs compare to all other electrodes also suggesting the smallest H-absorption energy which facile to hydrogen production through reducing strong chemical and/or electronic coupling at the PdMn-N_4_/CNTs catalyst surface^69^. For comparison, PdCo-N_4_/CNTs and PdCu-N_4_/CNTs catalysts are also employed for HER (Fig. S9a) and their corresponding Tafel analysis (Fig. S9b) which shown that the better HER activity at PdMn-N_4_/CNTs catalyst.

The long-term catalytic stability at the constant potential is also crucially important for an ideal HER electrocatalyst. Thus, the stability of PdMn-N_4_/CNTs, PdMn/CNTs and Pt/C electrodes using *i* vs. *t* measurement at an applied potential of *η*_*j*20_ = 80, 100 and 75 mV, respectively, in alkaline media for 30 h has been examined as in Fig. 5c. After 30 h, there is no significant degradation in current density at PdMn-N_4_/CNTs (only 11.4%) compare to PdMn/CNTs (31%) and Pt/C (43.4%), suggesting that the PdMn-N_4_/CNTs catalyst is superior stable for HER electrocatalysis for long time than those of PdMn/CNTs and Pt/C catalysts. After long-term test, the surface morphology is further observed by TEM analysis (Fig. S10). As can be seen in the TEM image of PdMn-N_4_/CNTs (Fig. S10a), the PdMn NPs is aggregated but the density of PdMn NPs still remains in higher amount compare to PdMn/CNTs (Fig. S10b) on the CNTs surface which indicating the better durability of PdMn-N_4_/CNTs catalyst probably due to the better attachment through N_4_-system.

In addition, the electrochemical impedance spectroscopy (EIS) analysis is finally employed to reveal the HER kinetics of those as-prepared catalysts through Nyquist plots in Fig. 5d. As shown in Fig. 5d, the smallest semicircle is produced at PdMn-N_4_/CNTs catalyst than PdMn/CNTs and Pd/CNTs which indicating a lower charge transfer resistance (*R*_ct_) and higher charge transport nature form bulk electrolyte at the PdMn-N_4_/CNTs surface. The *R*_ct_ is determined as ~52 Ω, 88 Ω and ~102 Ω for PdMn-N_4_/CNTs, PdMn/CNTs and Pd/CNTs, respectively). Therefore, the remarkably decreased *R*_ct_ leads to the faster HER catalysis on the PdMn-N_4_/CNTs composite electrode.

## Conclusion

The PdMn NPs anchored through N_4_-system onto CNTs has been synthesized using a facile wet-chemical method for electrochemical EOR and HER catalysis. The main catalytic site, Pd NPs are grown on Mn-N_4_/CNTs via epitaxial growth that consequence a highly efficient and stable EOR and HER using their morphological benefits such as the monolayer dispersion with smaller size of NPs, tight anchoring with the assistance of N_4_-system and electrical benefits such as higher ECSA and faster electron transfer ability. Superior activity, better reaction kinetics and higher durability during electrochemical EOR and HER than that of Pt/C electrode makes it favorable to the practical application in DEFCs and water splitting cells. Additionally, the synthesis strategy in this work can be extensively applied to prepare various low-cost and high-efficiency electrocatalysts.

## Supplementary information

Dataset 1
