# Supplementary material for: Carbon nanotubes-based PdM bimetallic catalysts through N4-system for efficient ethanol oxidation and hydrogen evolution reaction
Source: Sci Rep. 2019 Jul 30;9:11051. doi: 10.1038/s41598-019-47575-w (PMC6667450; doi:10.1038/s41598-019-47575-w)
Supplement: Supplementary file 1 — Dataset 1 [file 41598_2019_47575_MOESM1_ESM.docx]

Supporting information of

Carbon nanotubes-based PdM bimetallic catalysts through N_4_-system for efficient ethanol oxidation and hydrogen evolution reaction

Halima Begum, Mohammad Shamsuddin Ahmed, Dong-Weon Lee, Young-Bae Kim

Department of Mechanical Engineering, Chonnam National University, Gwangju, Republic of Korea. E-mail address: [ybkim@chonnam.ac.kr](mailto:ybkim@chonnam.ac.kr)

*Electrochemical characterization*

An ink of Pd/CNTs, PdMn/CNTs, or PdMn-N_4_/CNTs (1 mg mL^−1^ in ethanol) was prepared with ultrasonic agitation for 30 min in order to better dispersion. The prepolished glassy carbon electrode (GCE) of rotating ring disk electrode (RRDE) was then drop coated by a 10 μL portion of each ink separately. For the preparation of 20% Pt/C (E-TEK), the suspension of 1 mg mL^−1^ with 5 μL of Nafion (5%) in ethanol was dropped onto GCE. All electrochemical techniques were recorded using a CHI 700C electrochemical workstation (U.S.A.) three-electrode potentiostat and an EG&G Model 636 RDE system along with the CHI 700C used for RRDE measurements in high purity argon-purged (for at least 30 min) 1 M KOH solution at room temperature. A carbon-rod and a Ag/AgCl electrode were used as counter and reference electrode, respectively. The electrode potential was determined with respect to reversible hydrogen electrode (RHE) scale according to the Nernst equation: (*E*_RHE_=*E*_Ag/AgCl_ + 0.059 pH + *E*°0.197 V, at 25 °C).

*Instrumental Characterization*

The transmission electron microscopy and energy dispersive X-ray spectroscopy were carried out using a Tecnai 20 microscope at 200 kV, Crystal structure was examined by X-ray diffraction, which was carried out on a Rigaku D/max-2500, using filtered Cu Kα radiation. Detailed chemical compositions of the samples were analyzed by X-ray photoelectron spectroscopy using a VG multilab 2000 spectrometer (Thermo VG Scientific, Southend-on-Sea, Essex, UK) in an ultrahigh vacuum using an unmonochromatized Mg Kα (1253.6 eV) radiation source and a spherical section analyzer. Brunauer-Emmett-Teller (BET) surface area and pore size distribution using Barrett-Joyner-Halenda method were obtained through the nitrogen adsorption-desorption isotherm (BelsorpII mini, BEL Japan Inc.). X-ray absorption spectroscopy (XAS) was done by R-XAS (Rigaku, Japan)


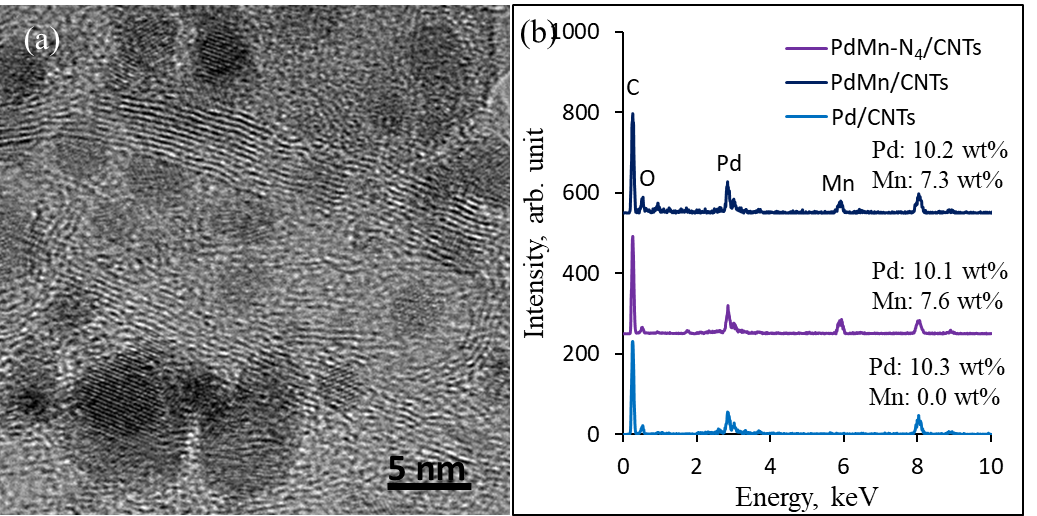


**Figure S1:** Enlarged TEM image of PdMn-N_4_/CNTs (a) and the EXS spectra of Pd/CNTs, PdMn/CNTs, and PdMn-N_4_/CNTs (b).


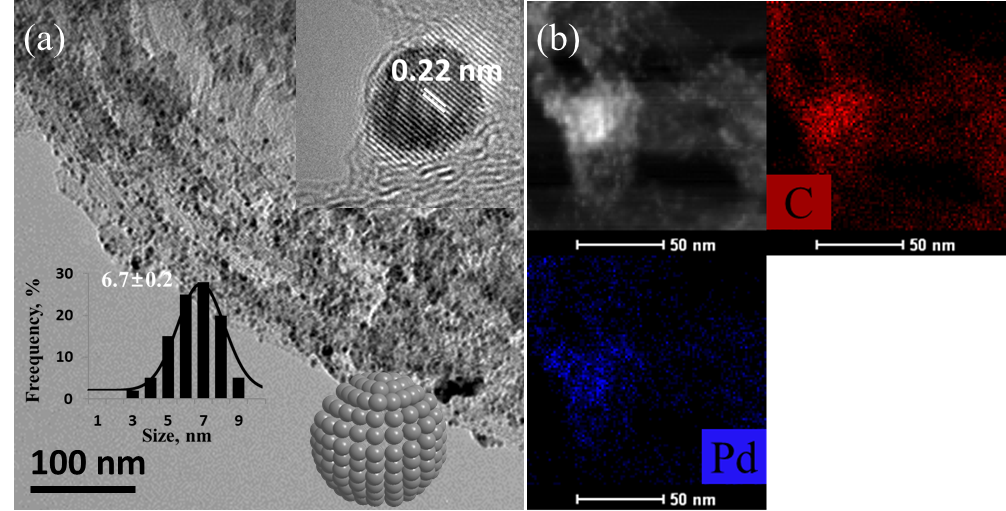


**Figure S2:** TEM image of Pd/CNTs, insets: a single Pd NPs showing lattice *d*-spacing (up) and simulated Pd NPs (down).

**
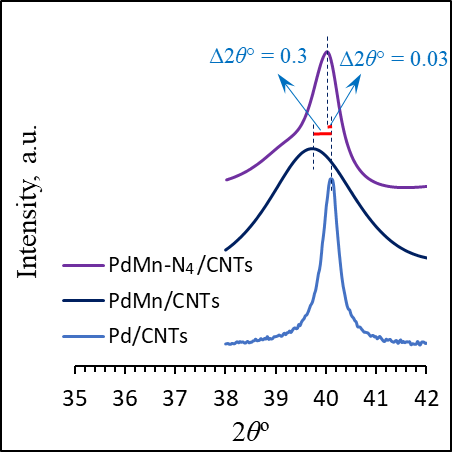
**

**Figure S3:** Enlarged XRD patterns of Pd/CNTs, PdMn/CNTs and PdMn-N_4_/CNTs.

**
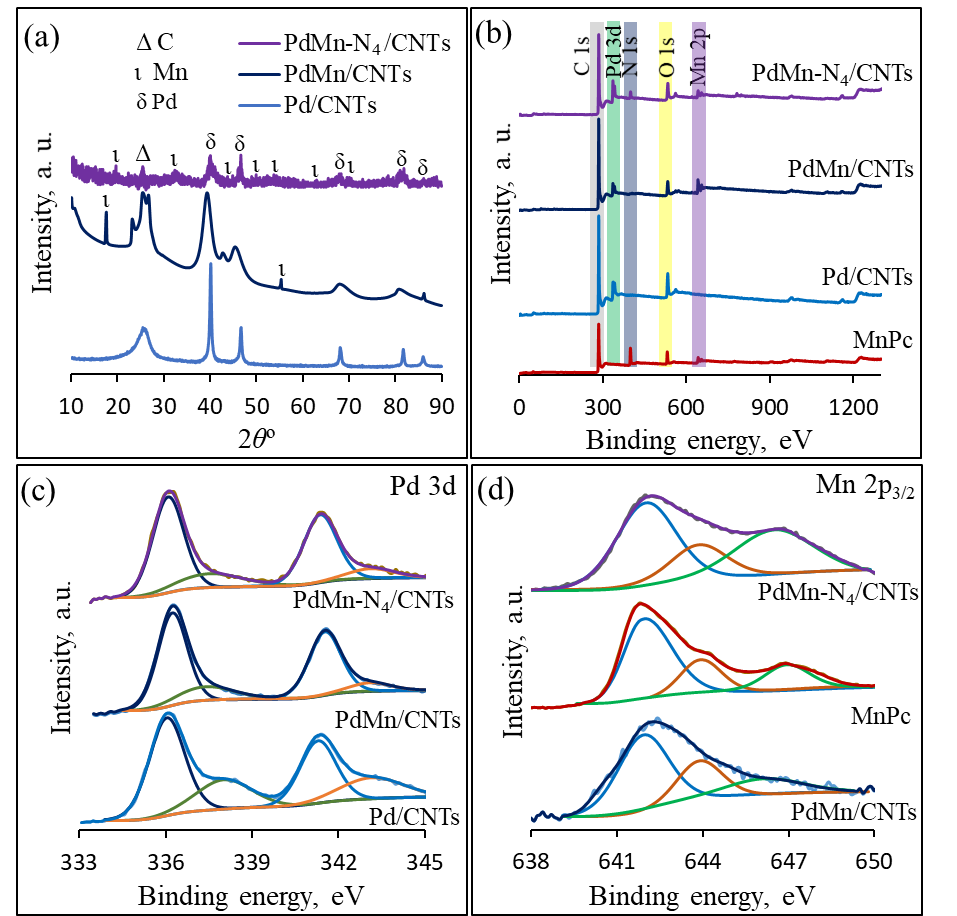
**

**Figure S4:** XRD patterns (a) and XPS spectra (b) of Pd/CNTs, PdMn/CNTs and PdMn-N_4_/CNTs; the XPS data of as purchased MnPc is used for comparison.


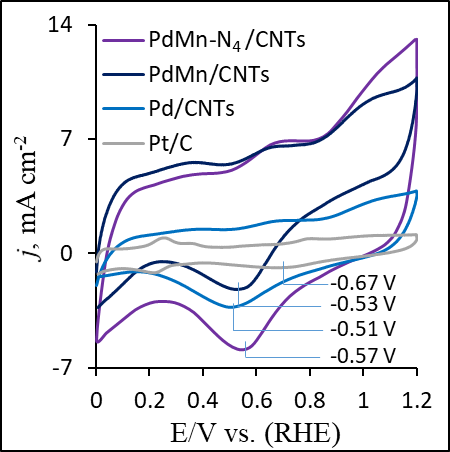


**Figure S5:** The CV curves in Ar-saturated 1 M KOH of Pd/CNTs, PdMn/CNTs, PdMn-N_4_/CNTs and Pt/C electrodes.


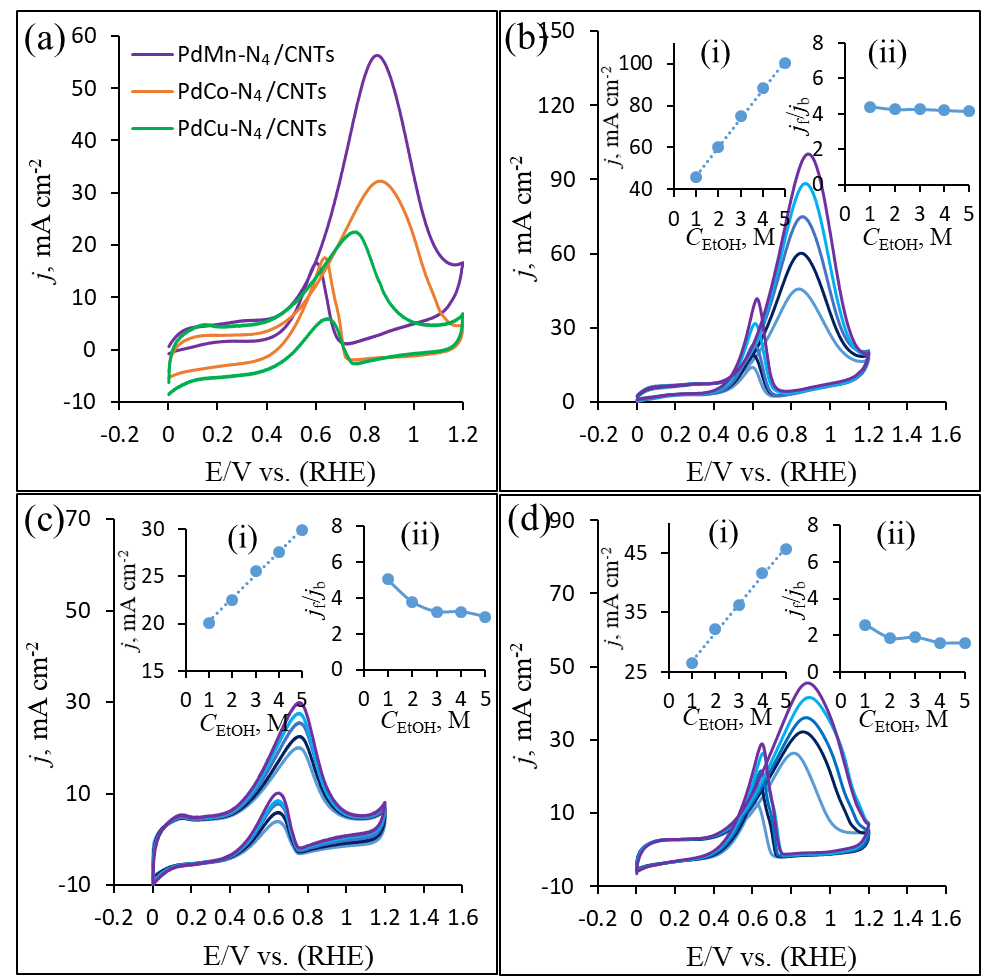


**Figure S6:** The CV curves recorded in Ar-saturated 1 M KOH with 2 M of ethanol on PdMn-N_4_/CNTs, PdCu-N_4_/CNTs and PdCo-N_4_/CNTs electrodes (a), the CV curves recorded in 1 to 5 M of ethanol addition at PdMn-N_4_/CNTs (b), PdCu-N_4_/CNTs (c) and PdCo-N_4_/CNTs (d) electrodes; insets: the plots of the $j_{f}$ (i) and $j_{f}$/$j_{b}$ (ii) vs. $C_{EtOH}$ for the respective electrodes.

**
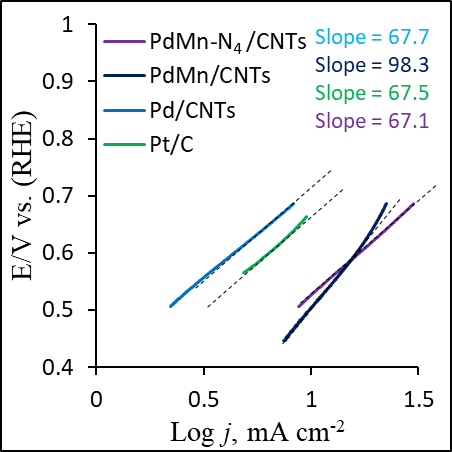
**

**Figure S7:** The Tafel plots derived from CVs of electrochemical EOR for Pd/CNTs, PdMn/CNTs, PdMn-N_4_/CNTs and Pt/C electrodes.


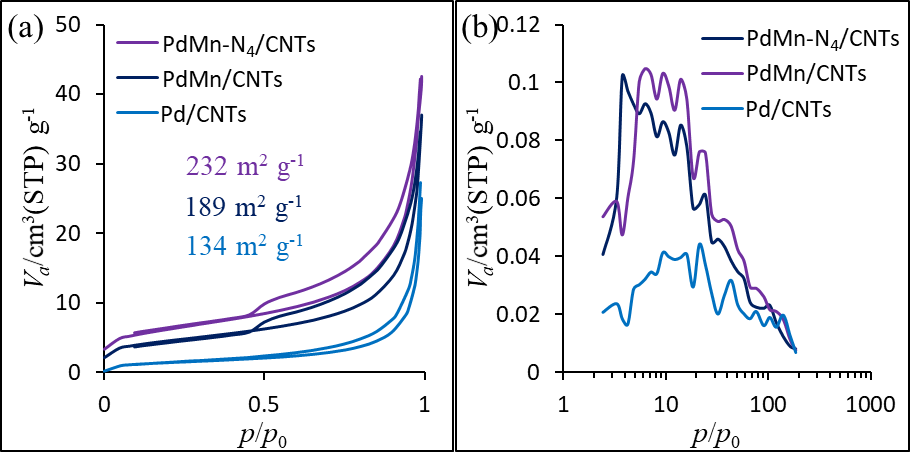


**Figure S8:** The nitrogen adsorption−desorption isotherms (a), pore size distribution (using BJH method) (b) of PdMn-N_4_/CNTs, PdMn/CNTs, and Pd/CNTs.


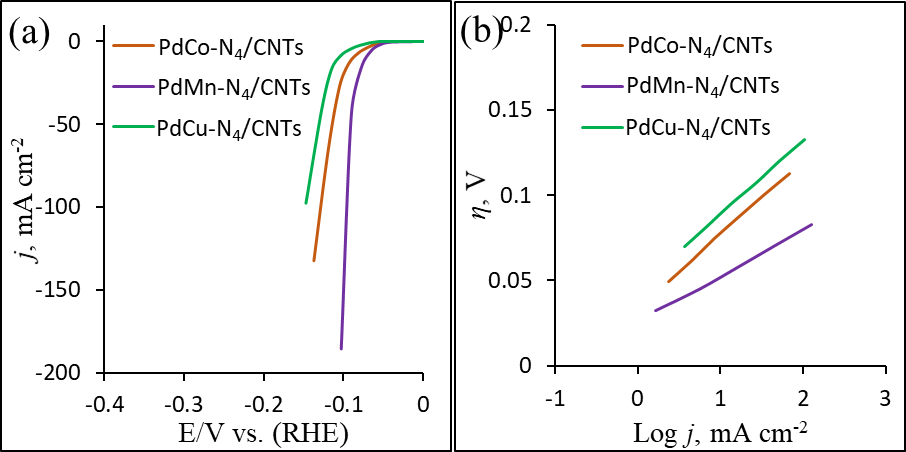


**Figure S9:** HER polarization curves at a scan rate of 5 mV s^−1^ (a) and the corresponding Tafel plots (b) of PdMn-N_4_/CNTs, PdCo-N_4_/CNTs and PdCu-N_4_/CNTs electrodes in 1 M KOH solution.


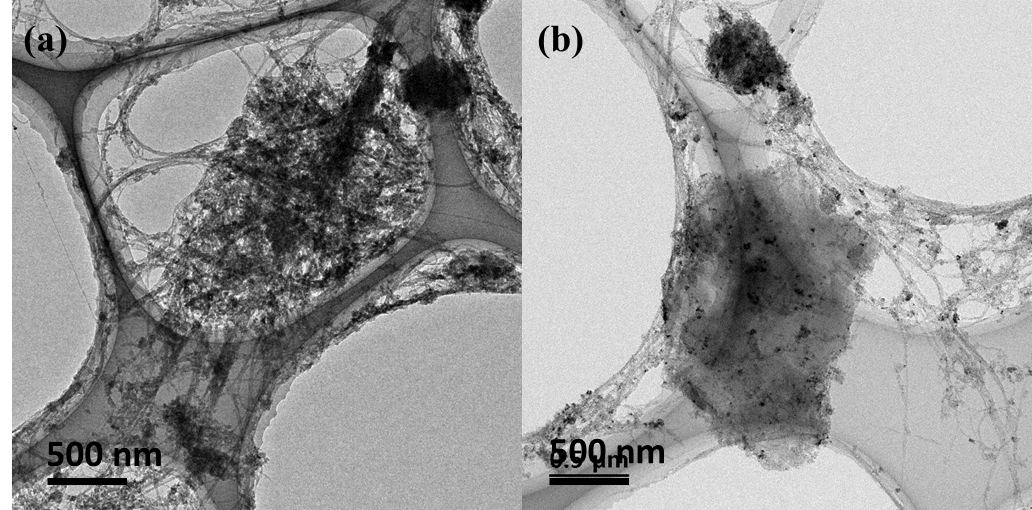


**Figure S10:** TEM images of PdMn-N_4_/CNTs (a) and PdMn/CNTs (b) after 30 hours of HER in 1 M KOH solution.
